# Impact of synthetic data on training a deep learning model for lesion detection and classification in contrast-enhanced mammography

**DOI:** 10.1117/1.JMI.12.S2.S22006

**Published:** 2025-04-28

**Authors:** Astrid Van Camp, Henry C. Woodruff, Lesley Cockmartin, Marc Lobbes, Michael Majer, Corinne Balleyguier, Nicholas W. Marshall, Hilde Bosmans, Philippe Lambin

**Affiliations:** a Maastricht University, GROW – Research Institute for Oncology and Reproduction, Department of Precision Medicine, Maastricht, The Netherlands; b KU Leuven, Division of Medical Physics & Quality Assessment, Department of Imaging and Pathology, Leuven, Belgium; c Maastricht University Medical Centre+, GROW – Research Institute for Oncology and Reproduction, Department of Radiology and Nuclear Medicine, Maastricht, The Netherlands; d UZ Leuven, Department of Radiology, Leuven, Belgium; e Zuyderland Medical Center, Department of Medical Imaging, Sittard-Geleen, The Netherlands; f Université Paris Saclay, Institut Gustave Roussy, Department of Imaging, Villejuif, France

**Keywords:** contrast-enhanced mammography, synthetic data, deep learning, radiomics, microcalcification cluster

## Abstract

**Purpose:**

Predictive models for contrast-enhanced mammography often perform better at detecting and classifying enhancing masses than (non-enhancing) microcalcification clusters. We aim to investigate whether incorporating synthetic data with simulated microcalcification clusters during training can enhance model performance.

**Approach:**

Microcalcification clusters were simulated in low-energy images of lesion-free breasts from 782 patients, considering local texture features. Enhancement was simulated in the corresponding recombined images. A deep learning (DL) model for lesion detection and classification was trained with varying ratios of synthetic and real (850 patients) data. In addition, a handcrafted radiomics classifier was trained using delineations and class labels from real data, and predictions from both models were ensembled. Validation was performed on internal (212 patients) and external (279 patients) real datasets.

**Results:**

The DL model trained exclusively with synthetic data detected over 60% of malignant lesions. Adding synthetic data to smaller real training sets improved detection sensitivity for malignant lesions but decreased precision. Performance plateaued at a detection sensitivity of 0.80. The ensembled DL and radiomics models performed worse than the standalone DL model, decreasing the area under this receiver operating characteristic curve from 0.75 to 0.60 on the external validation set, likely due to falsely detected suspicious regions of interest.

**Conclusions:**

Synthetic data can enhance DL model performance, provided model setup and data distribution are optimized. The possibility to detect malignant lesions without real data present in the training set confirms the utility of synthetic data. It can serve as a helpful tool, especially when real data are scarce, and it is most effective when complementing real data.

## 1 Introduction

Breast cancer is a heterogeneous disease with differences in tumors and among cancer phenotypes that encompass various pathologies. This diversity requires precise methods for detection and characterization. These pathologies include space-occupying masses that have grown to replace healthy tissue and microcalcification clusters consisting of small calcium deposits.^1^ Although not inherently cancerous, these clusters may indicate an underlying malignant process, necessitating their evaluation for early detection and characterization.^2^ Several breast imaging techniques exist for cancer screening and diagnosis with digital mammography (DM) being the most widely utilized.^3^ DM is effective in imaging various pathologies but correctly characterizing all suspicious lesions and detecting subtle or small abnormalities remains challenging.

An innovative and evolving advancement in breast imaging is contrast-enhanced mammography (CEM), which is increasingly being introduced in hospitals. It shows promising potential for improved sensitivity and has a higher negative predictive value than traditional DM,^4^ particularly for dense breasts.^5^ Similar to breast magnetic resonance (MRI) imaging, CEM improves both sensitivity and specificity^6^ by utilizing a contrast agent administered to the patient, more precisely an iodine-based contrast agent for CEM and gadolinium-based MRI. The CEM contrast uptake is captured by acquiring a low-energy image and a high-energy image, below and above the K-edge of iodine, respectively. Through image subtraction and image processing, a recombined image is created that highlights these areas of contrast uptake. Concurrently, the low-energy image, which is equivalent to DM,^7^^,^^8^ offers better visualization of the breast architecture as well as lesions that do not take up contrast.

Given their complementary nature, the detection of the different breast cancer pathologies should thus utilize both low-energy and recombined images. The enhancing lesions are commonly characterized as space-occupying masses, in which cancerous regions often develop neovascular structures, facilitating their detection in the recombined image via iodine uptake. Conversely, the small, non-enhancing microcalcifications are primarily visible in the low-energy image. However, some types of mass lesions may develop in the vicinity of a microcalcification cluster, leading to local enhancement as well as increased conspicuity for cancer.^9^^,^^10^

The challenges in detecting microcalcifications and the overall need for increased sensitivity and specificity highlight the necessity for automation and/or computer-aided decision-making in breast imaging. Automation can potentially provide radiologists with valuable insights regarding the presence of malignancies and the need for further clinical investigation.^11^ Previous studies have applied machine learning models utilizing handcrafted radiomics features^12^^,^^13^ to classify suspicious breast lesions in both DM and CEM.^14^ In addition, deep learning (DL) techniques have demonstrated proficiency in performing lesion detection and classification.^15^ Both offer support to radiologists in diagnostic decision-making. Despite their overall high accuracy, current models for CEM have not adequately considered the difference between enhancing masses and lesions primarily characterized by the presence of microcalcifications. Our research aims to fill this gap by achieving high performance across all lesion types.

Previous work by Beuque et al.^16^ has established a lesion characterization pipeline for CEM and obtained a promising performance. In this work, the development of this comprehensive model for characterizing all lesion types within a single CEM exam was continued. Alongside a DL model for detection and classification, a handcrafted radiomics classifier was trained in parallel. Due to the limited availability of CEM cases featuring a microcalcification cluster, we opted to generate synthetic data with simulated microcalcification clusters and enhancement. With this novel approach, we aimed to provide the means for data augmentation to increase the training set, specifically for difficult-to-detect and rare lesions. As a whole, this study will investigate the hypothesis that incorporating synthetic data in our DL model training can enhance the sensitivity for detecting all types of suspicious breast lesions without compromising the classification performance.

## 2 Materials and Methods

### 2.1 Clinical Data

This study utilized two retrospectively collected datasets with post-processed CEM images. The first consisted of images from Maastricht University Medical Centre (MUMC) (Maastricht, the Netherlands), acquired on a Senographe Essential system with SenoBright (GE Healthcare, Buc, France) between 2013 and 2018, and first described by Beuque et al.^16^ The second consisted of images from Gustave Roussy Institute (GR) (Villejuif, France) acquired on Senographe Essential and Senographe Pristina systems with SenoBright (GE Healthcare, Buc, France) between 2015 and 2019. Both datasets consisted of recombined and post-processed low-energy images acquired in craniocaudal (CC) and mediolateral oblique (MLO) views. The requirement for informed consent was waived by the respective institutional review boards: approval no. METC 2019–0995 for the MUMC dataset and approval no. 2022–140 for the GR dataset.

**Fig. 1:**
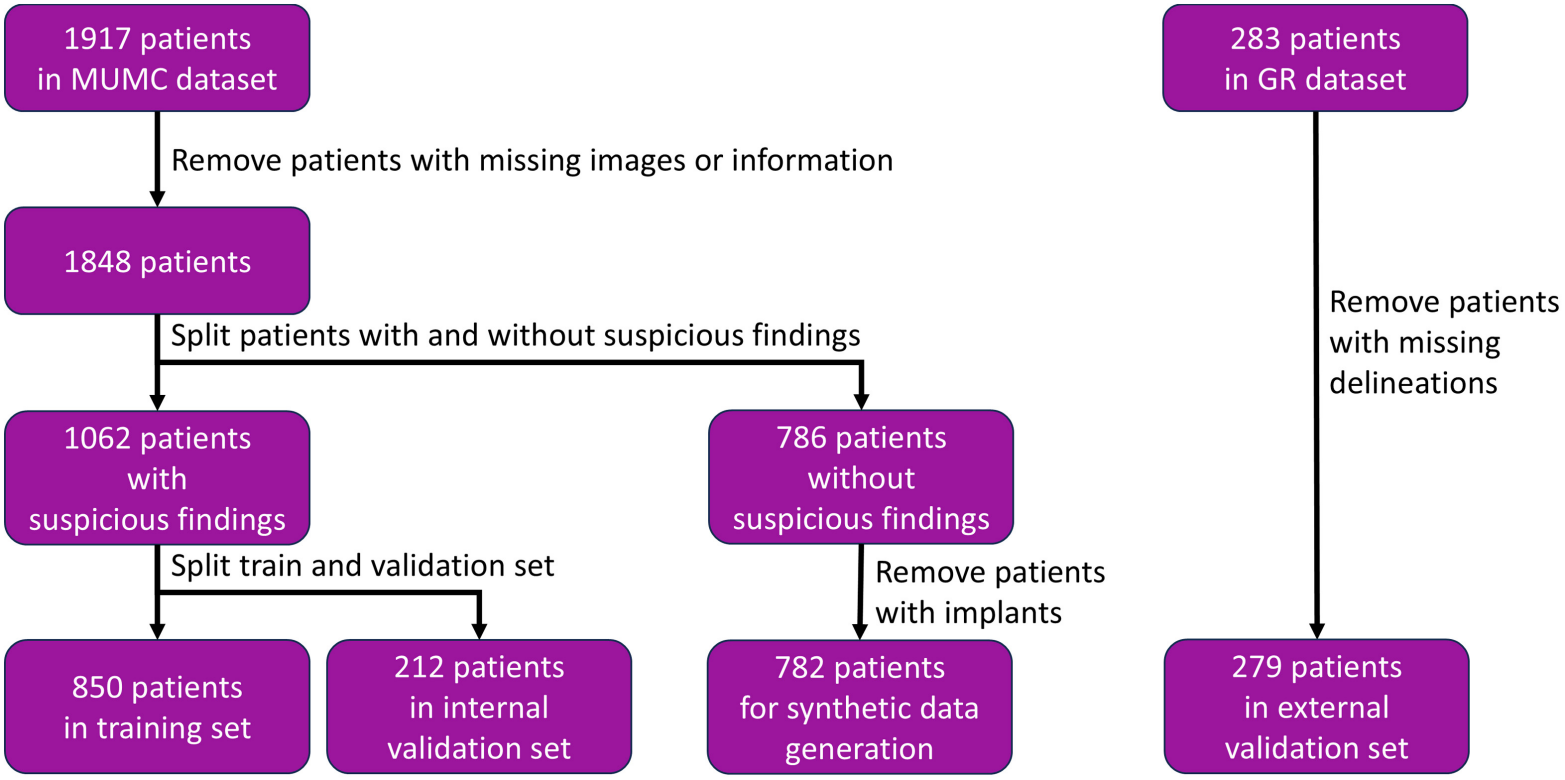
Overview of the clinical data.

The MUMC dataset comprised images of 1917 patients with and without suspicious findings in CEM. Details on how these data were divided and used in this study are depicted in Fig. 1. Images of the 782 patients without any suspicious findings in CEM were used to generate the synthetic cases with simulated lesions inserted. Patients with implants were removed because the contrast and intensity of the implants in the image could negatively affect the synthetic data generation approach.

The predictive models could only be trained on images with suspicious findings present. Therefore, images of breasts containing suspicious findings were used for training (80%, 850 patients) and internal validation (20%, 212 patients), split at the patient level. The images of breasts containing suspicious findings in CEM in the GR dataset (279 patients) were used as an external validation set. This set assessed the accurate detection and classification of lesions of the trained DL and handcrafted radiomics models.

### 2.2 Synthetic Data Generation

**Fig. 2:**
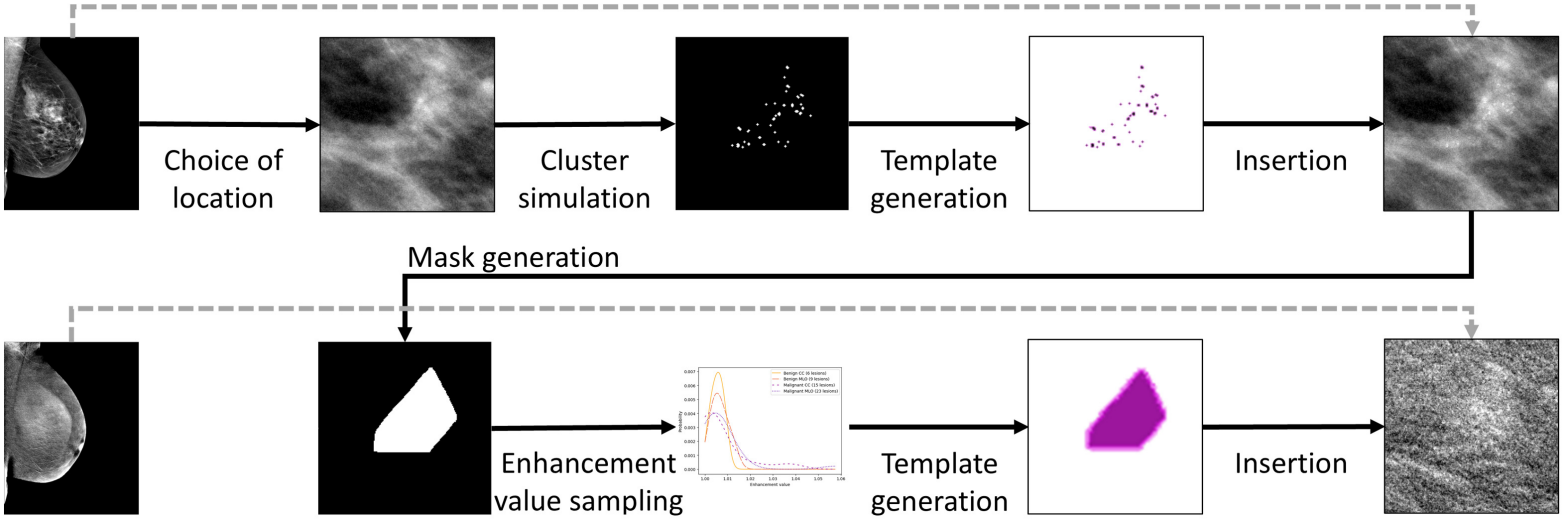
Pipeline to generate synthetic data with the process to simulate and insert a microcalcification cluster in a low-energy image in the upper line and the process to simulate associated enhancement in the corresponding recombined image in the bottom line.

To increase the data available to train a DL model, synthetic data were generated.^17^ The pipeline is summarized in Fig. 2, which demonstrates how simulated microcalcification clusters were inserted into low-energy images whereas enhancement was simulated in the corresponding recombined images.

#### 2.2.1 Microcalcification cluster simulation

In previous work, we established methods to generate large sets of unique microcalcification cluster models.^18^^,^^19^ This work included an approach that utilized a radiomics analysis^12^ of local breast tissue textures to locate a plausible location for insertion and identify candidate regions for calcifications at this location. This approach was applied to generate unique two-dimensional (2D) models of clusters specific to the low-energy images of each view (CC or MLO) from patients without suspicious findings in the MUMC dataset.^17^

In brief, radiomics feature values were computed in a grid of non-overlapping 200×200  pixel cells covering the breast region to locate denser regions with greater structural variety.^19^ Ranking the grid cells with the highest or lowest values (depending on the feature) resulted in the highest-scoring cell which was considered the most plausible location for insertion. Within this cell, a binary mask was created by multiplying the output mask of a Frangi filter^20^ with a mask obtained with an intensity threshold.^19^ All connected regions of value 1 in this mask were considered candidate regions for calcifications based on their morphology and intensity.

Next, relevant candidate regions for calcifications were further selected according to circularity and size criteria.^19^ For typically benign clusters these ranges were [0.5, 1.0] and [0.3, 1.2 mm], respectively, preserving the larger and more circular calcifications.^21^ For malignant clusters, any value of circularity was allowed and a range of [0.1, 0.5 mm] was imposed on the size to preserve the small, irregularly shaped calcifications.^22^ To choose which candidate regions to add as calcification to the cluster, a finer grid was used to locate the 10×10  pixel sub-cell with the highest contrast. From this sub-cell, cluster creation was initialized by adding all candidate regions within the sub-cell to the cluster as calcifications. The search region was iteratively enlarged by incorporating the neighboring sub-cells and adding their calcifications to the cluster until a predetermined number of calcifications was reached. This number was chosen between 10 and 20 for a benign cluster and between 20 and 40 for a malignant cluster. This process yielded a 2D binary cluster model with the same pixel resolution as the low-energy image, with unity denoting the presence of calcifications, and zero for the background.^19^

#### 2.2.2 Cluster insertion with a hybrid simulation framework

As it was previously found that calcifications are not clearly visible in the recombined image as they do not take up contrast agents themselves,^18^ the resulting cluster model was only inserted in the post-processed low-energy image. To do so, the 2D binary cluster model was converted to a template with template values that mimic how the ray-tracing and processing of the acquisition system would present the calcifications.^19^ Degrading factors such as scatter and blurring were applied to the template, converting it to a final version ready for insertion by multiplication with the low-energy image.^23^^,^^24^ Within this template, background pixels had a value of 1.0 to preserve the original image upon multiplication, and the calcifications had pixel values greater than 1.0. In the post-processed low-energy image with an inverted lookup table, calcifications inserted after multiplication thus appeared brighter (whiter) due to their higher intensity values.

Automatic mask generation was included in the workflow to obviate the need for manual segmentation of each synthetic case. To generate a mask, a convex hull was defined around all the inserted calcifications, for which the exact location was known from the template. The convex hull could exhibit sharp corners, and these were smoothed by a technique similar to the corner-cutting method described by Chaikin.^25^ Rather than cutting the corners and thus reducing the surface area, this technique instead enlarged the contour by adding points outside of the hull. A comprehensive explanation and visual examples of this technique can be found in the Supplementary Material. A final dilation step was applied to expand the mask further, increasing the resemblance to delineations performed by radiologists, who often include a margin of the perilesional tissues in their annotations of calcifications.

#### 2.2.3 Enhancement simulation

The mask generation also enabled enhancement to be simulated within the region surrounding the inserted cluster. For this purpose, pixel values in the recombined image within the mask were adjusted using a factor derived from the enhancement occurring in real lesion cases of the MUMC dataset.^17^ This factor was computed by measuring the mean pixel value (${\text{meanPV}}_{\text{lesion}}$) within real delineated lesion regions. In addition, perilesional and background regions of the equivalent area were determined. To this end, the initial real lesion mask was incrementally dilated, first to contain a number of pixels double that of the lesion mask and second to triple the initial pixel count. The region created by the first dilated mask minus the lesion mask thus represented the perilesional region, and the second dilation minus the lesion mask and the perilesional mask represented a background region equivalent to the original mask for the lesion. The mean pixel value for this background region (${\text{meanPV}}_{\text{background}}$) was measured and the enhancement value was calculated as the ratio of ${\text{meanPV}}_{\text{lesion}}$ to ${\text{meanPV}}_{\text{background}}$ for each image.

**Fig. 3:**
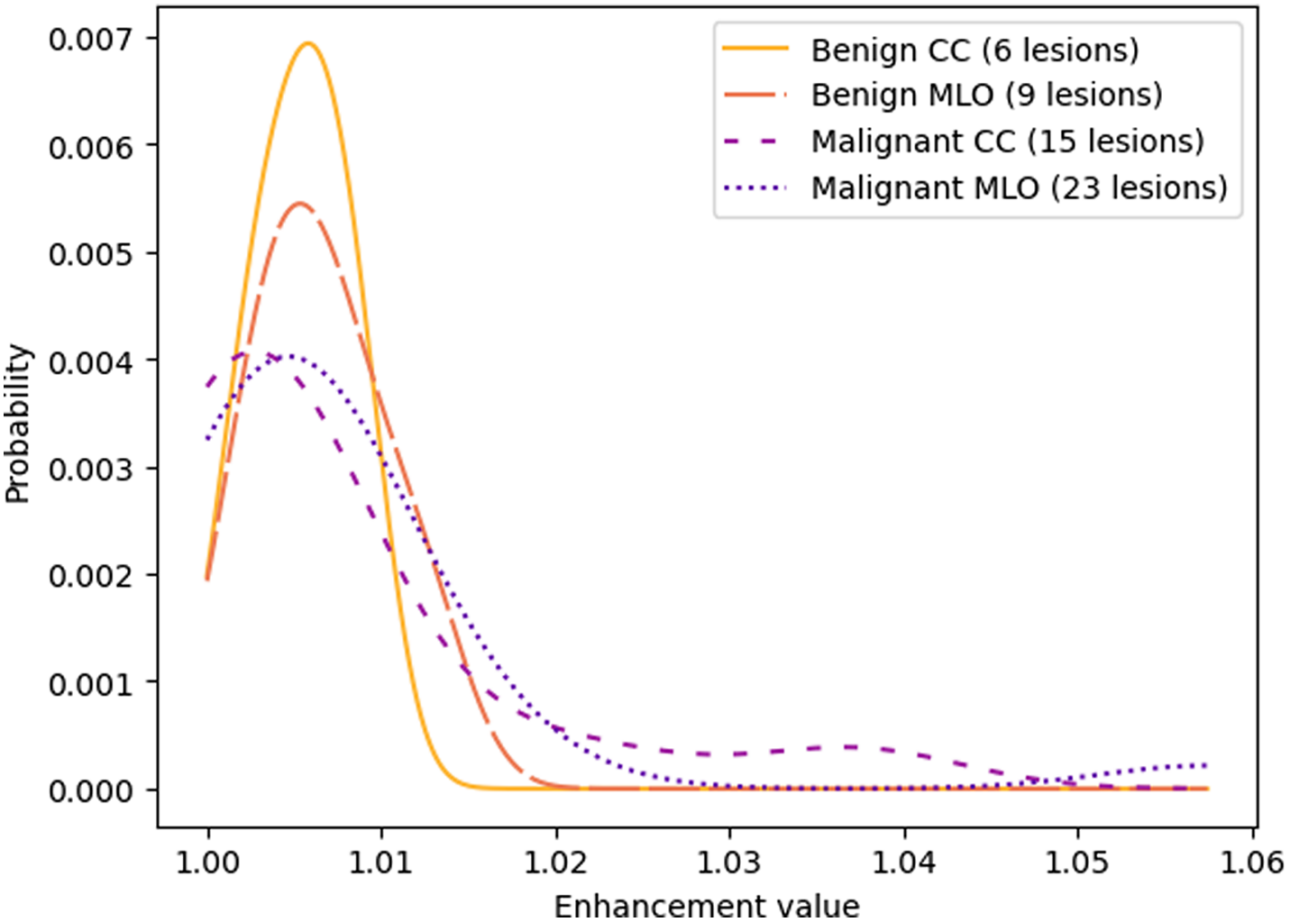
Normalized probability distributions of the enhancement values of non-detected lesions with calcifications.

These enhancement values ranged from 1.0, indicating no enhancement in the recombined image, up to ∼1.06 for the most enhancing lesions. The probability density function of these values was approximated with a Gaussian kernel and normalized such that the weighted sum of all probabilities was equal to 1, as displayed in Fig. 3 for subsets of different mammographic views (CC versus MLO) and different lesion types (malignant versus benign clusters). For this work, the cases considered were limited to those for which the presence of microcalcifications was established by the description of the radiologist. The aim of this work was to improve the sensitivity of a DL model further described in Sec. 2.3, especially for difficult-to-detect lesions. Therefore, the measured enhancement values were constricted to lesions that were not detected by the DL model trained with the complete real training set (see Sec. 2.3.1).

For each cluster inserted in the low-energy image, an enhancement value between 1.00 and 1.06 was sampled from the probability distribution for the specific type of cluster (benign or malignant) and the view (CC or MLO). The mask generated with the method from Sec. 2.2.2 was transformed to create a template with pixel values equal to the enhancement value within the lesion region and a value of 1.0 elsewhere. To simulate a gradual decrease in enhancement toward the edge of the lesion and ensure more realistic blending with the surrounding tissue, a Gaussian smoothing kernel with a standard deviation of 5.0 was applied to the template. Finally, this mask was multiplied with the recombined image at the location corresponding to the insertion of the cluster. This increase in resulting pixel values effectively simulated enhancement in the recombined image.

### 2.3 Detection and Classification Models

**Fig. 4:**
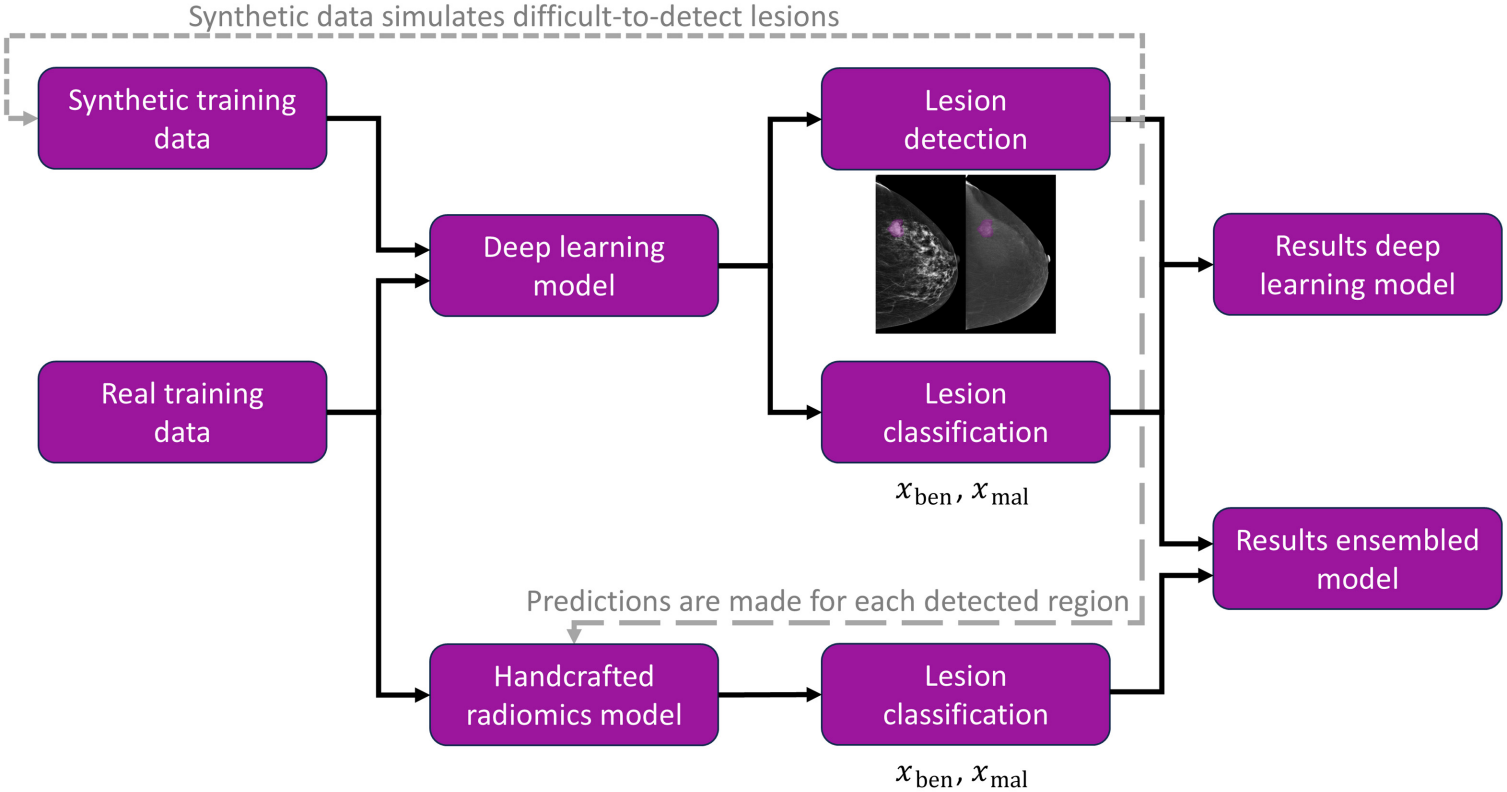
Outline of the predictive models training.

This section presents two models for detecting and classifying lesions in CEM data, a DL model and an XGboost classifier^26^ utilizing handcrafted radiomics,^12^ and the combination of their predictions in an ensemble model. Figure 4 depicts the outline of the model training workflow, with real and/or synthetic data. Each model generates predictions, and these can be combined in an ensembled model, in which case, the handcrafted radiomics model predicts a class for each region of interest (ROI) detected.

#### 2.3.1 Training data setup

With real and synthetic data available, multiple training data setups were constructed. First, subsets of the real training set were obtained by randomly sampling patients while maintaining the same ratios between benign and malignant lesions and between masses and clusters. The different subsets gradually doubled the number of cases, resulting in sets equal to 5%, 10%, 20%, 40%, and 80% of the complete real training set.

Similarly, four synthetic training sets of varying sizes were created to investigate the impact of the amount of synthetic data added during training. During their construction, it was ensured each synthetic set preserved the ratio of benign microcalcification clusters to malignant microcalcification clusters of the complete real training set. A first, small, synthetic set was equivalent to 20% of the number of clusters in the complete real training set. Similarly, the second and third synthetic sets were equivalent to 100% and 200% of the number of clusters in the complete real training set, respectively. The last setup addressed the class imbalance between masses and clusters in the complete real training set. Considering the difference between the number of patients with solely a mass present and those with a microcalcification cluster, this imbalance removal setup was equivalent to 317% of the number of clusters in the complete real training set.

The DL model was then trained with each of the real training subsets, and each of the constructed synthetic sets individually. In addition, real and synthetic training sets were combined to create mixed training data setups. For a complete table with the number of images in each setup, we refer to Supplemental 2 in the Supplementary Material. The number of masses was never increased; thus, the addition of synthetic data always altered the ratio between masses and clusters. Evaluation of all models was performed on the same internal and external validation sets containing solely real data. These equal validation sets allowed for an accurate comparison.

#### 2.3.2 Deep learning model

The method for pre-processing the training and validation data has been previously described by Beuque et al.^16^ In this work, only the creation of the red, green, blue (RGB) image was altered^27^: the first channel consisted of the contrast limited adaptive histogram equalization (CLAHE)-filtered low-energy image, the second of the original low-energy image, and the third of the CLAHE-filtered recombined image. This combination puts more weight on the low-energy image which better visualizes calcifications, the types of lesions that were previously found to be the most difficult to detect.^16^ The resulting RGB image was saved in portable network graphics (PNG) format to avoid data loss through compression.

Detection and classification were performed using a mask R-CNN architecture^28^ with a ResNet-50-FPN backbone, which was newly implemented for this study from the PyTorch library. The network was trained to differentiate among four classes: benign masses, malignant masses, benign clusters, and malignant clusters. This classification was found to better detect all lesion types than a model differentiating solely between benign and malignant lesions.^27^ The weights were pre-trained on the COCO dataset.^29^ Further training on the training set was conducted for 30 epochs, with an Adam optimizer, a learning rate of $10^{-5}$, and a batch size of 2. For every set of training data, training was performed in exactly the same setup. The optimal epoch was considered to be the one yielding the lowest loss on the internal validation set, without any other change in hyperparameters.

The output of the mask R-CNN consisted of a bounding box surrounding a segmentation mask. For each box, the highest prediction score among all classes was returned along with its corresponding class label. This prediction score pred_score, ranging from 0.0 to 1.0, denoted a measure of the model’s confidence in the predicted class label. In this study, a lesion was considered detected by the DL model if the Intersection over Union (IoU) between the real mask and the predicted segmentation mask was higher than 0.1, and the prediction score was higher than 0.1.^16^

#### 2.3.3 Handcrafted radiomics model

As well as the mask R-CNN model, a handcrafted radiomics model for benign–malignant classification was trained. Details about the implementation and feature selection can be found in previous work.^16^ In this work, radiomics features were extracted from the annotated lesion ROIs in both the low-energy image and the recombined image and then combined. Feature reduction consisted of removing correlated features via Spearman’s correlation coefficient. The remaining features were further eliminated with cross-validation to find an optimal number for XGBoost classifier^26^ training. This XGBoost classifier was then trained with the real internal training set.

During inference, radiomics features were extracted from the ROIs segmented by the DL models. For each of the ROIs, the optimally tuned and trained XGBoost model then made a classification considering the extracted feature values. The predicted probability values $P_{\mathrm{HR}}(x=\text{ben})$ and $P_{\mathrm{HR}}(x=\text{mal})$ for the benign and malignant class, respectively, ranged between 0.0 and 1.0.

#### 2.3.4 Prediction analysis

Although the handcrafted radiomics classifier returned probabilities exhibiting a binomial distribution, the DL model returned a class label and a corresponding prediction score pred_score between 0.0 and 1.0. To combine the predictions of both trained models, the prediction score of the DL model was modified to align it with the binomial distribution of the handcrafted radiomics model. If the DL model had categorized an ROI in any of the benign classes, the benign probability value was $p_{\mathrm{DL}}(x=\text{ben})=0.5*\mathrm{pred}\_\text{score}+0.5$ and the malignant $p_{\mathrm{DL}}(x=\text{mal})=1.0-p_{\mathrm{DL}}(x=\text{ben})$. If the DL had categorized the ROI in any of the malignant classes, this modification was inverted: $p_{\mathrm{DL}}(x=\text{mal})=0.5*\mathrm{pred}\_\text{score}+0.5$ and $p_{\mathrm{DL}}(x=\text{ben})=1.0-p_{\mathrm{DL}}(x=\text{mal})$. This modification ensured that the benign and malignant classification probability values of both the DL model and the radiomics model ranged between 0.0 and 1.0, with the value for the predicted class being higher than 0.5. The performance of the DL model for detection and classification was compared with the results of the ensembled model averaging the DL and radiomics probability values. As the main focus was the capacity of the models to distinguish benign from malignant lesions, both DL class labels for benign masses and benign clusters were mapped to “benign” for further analysis and similar for “malignant.”

A predicted ROI was considered a correct detection if it was both overlapping with a true lesion ROI (i.e., an IoU greater than 0.1) and was correctly categorized in the benign versus malignant classes. Therefore, a threshold of 0.5 for the classification probability value was chosen to determine benign and malignant categorized predictions. To compute precision, the correct detections were compared with all predicted ROIs, comprising the correct detections as well as the ROIs that either did not overlap with a true lesion ROI or overlapped but were wrongly classified. To compute sensitivity, the correct detections were compared with all true lesion ROIs, comprising those overlapping with correct detections as well as the true ROIs for which none of the predicted ROIs had an IoU greater than 0.1 or for which the overlapping predicted ROIs had a wrong class label.

For the classification stage, all predicted ROIs of the detection stage were further analyzed. For predicted ROIs with an IoU greater than 0.1, the correct class was the class of the overlapping true lesion ROI. For predicted ROIs with an IoU smaller than 0.1, we considered it correctly classified if the predicted class was benign because a model was considered less optimal if it wrongly predicted the presence of a malignant ROI than of a benign ROI. The true positive rate of ROI classification was plotted against the false-positive rate in the receiver operating characteristic (ROC) curve. To assess the performance of the classification models, the area under this ROC curve (AUC) was considered.

For all metrics, 95% confidence intervals (CIs) were computed using bootstrapping. This process computed the performance 2000 times by repeatedly sampling a number of patients from the validation set which was equal to the number of patients in the set at hand yet allowing the same patient to occur more than once. In addition, for each real subset, it was determined which combination with synthetic data achieved the highest detection sensitivity of malignant lesions. The metrics for training with solely the real subset and for training with the optimal combination with synthetic data were compared: the mean difference and the 95% CIs of the difference among metrics were computed. A similar analysis was performed for each synthetic training set and its optimal combination with a real subset.

## 3 Results

### 3.1 Clinical Data

The complete MUMC dataset comprised 1917 patients, although information or images were incomplete for some patients. The available information comprised clinical factors (detailed by Beuque et al.^16^ and summarized in Supplemental 3 in the Supplementary Material, as well as lesion delineations drawn by an expert radiologist and descriptions provided by the radiology report). Pathology outcomes for biopsy provided a definite benign or malignant classification status. This comprehensive information facilitated the categorization of lesions into two groups: lesions with a microcalcification cluster present, and those without, further referred to as “masses” for brevity.

Among the 283 patients in the GR set, 279 had one or more suspicious lesions present with images and delineations available, which were included in the external validation set (Fig. 1). Similarly, clinical information and biopsy outcomes provided a categorization of benign and malignant lesions. Due to the limited radiological information available, no distinction could be made among lesions consisting solely of (enhancing) masses or those with calcifications present.

### 3.2 Synthetic Data Generation

Microcalcification clusters and enhancement were simulated and inserted in each of the 782 patients without suspicious findings in the MUMC dataset. The first simulation specified parameter values of typically benign lesions, and the second simulation created malignant lesions.^19^ If insufficient candidate calcification regions met the specified size and circularity conditions, no simulation for this view was performed. As clusters were only simulated in one 2D mammographic projection, the cluster models inserted in CC and MLO views of the same breast were different. However, this did not interfere with the training goal as the DL model was trained on an image level. Therefore, it was only taken into consideration that both views for the same breast should have either a benign or a malignant cluster.

**Fig. 5:**
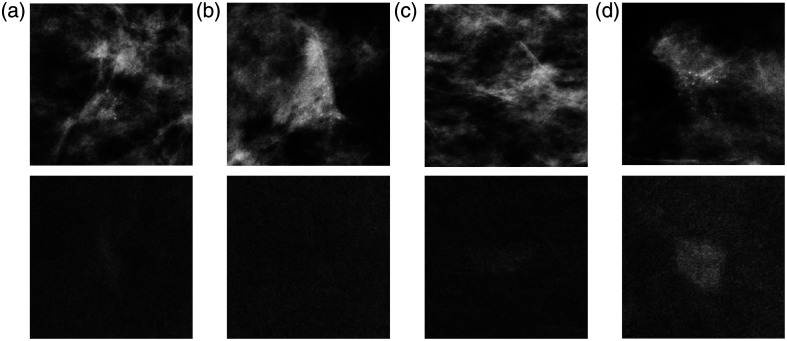
Examples of synthetic data with microcalcification clusters simulated in the low-energy image (upper line) and enhancement in the recombined image (lower line). Cases (a) and (b) are typically benign clusters. Cases (c) and (d) are typically malignant clusters.

Figure 5 shows the examples of simulated clusters and enhancement inserted in lesion-free images of the MUMC dataset. All microcalcification cluster architectures tended to follow the structures exhibited by the breast tissue. Validation studies had previously assessed the realism of the simulated microcalcification clusters.^19^ In addition, the probability distributions of the enhancement in all simulations closely followed those of Fig. 3 for the specific type of cluster (benign or malignant) and the view (CC or MLO). As expected from these distributions, the malignant lesions [Figs. 5(c) and 5(d)] show higher levels of enhancement than the benign lesions [Figs. 5(a) and 5(b)].

### 3.3 Detection and Classification Models

#### 3.3.1 Training data setup

The selection and combination of the data yielded 34 distinct training data setups: 6 with different amounts of solely real data, 4 with different amounts of solely synthetic data, and 24 with a combination of real and synthetic data. The total number of images in each set can be found in Supplemental 2 in the Supplementary Material. The DL model was trained for each setup on a Nvidia Geforce RTX 2080 Ti graphical processing unit. The training of 30 epochs took between 52 min and 11 h, depending on the size of the training sets. For most setups, minimal loss on the internal validation set occurred at epoch 8. The only exceptions were the training with 5% of real data (epoch 16), 5% of synthetic data (epoch 4), 200% of synthetic data (epoch 12), and the imbalance-removal synthetic set (epoch 12). Metrics were calculated as described in Sec. 2.3.4.

#### 3.3.2 Deep learning model performance

**Fig. 6:**
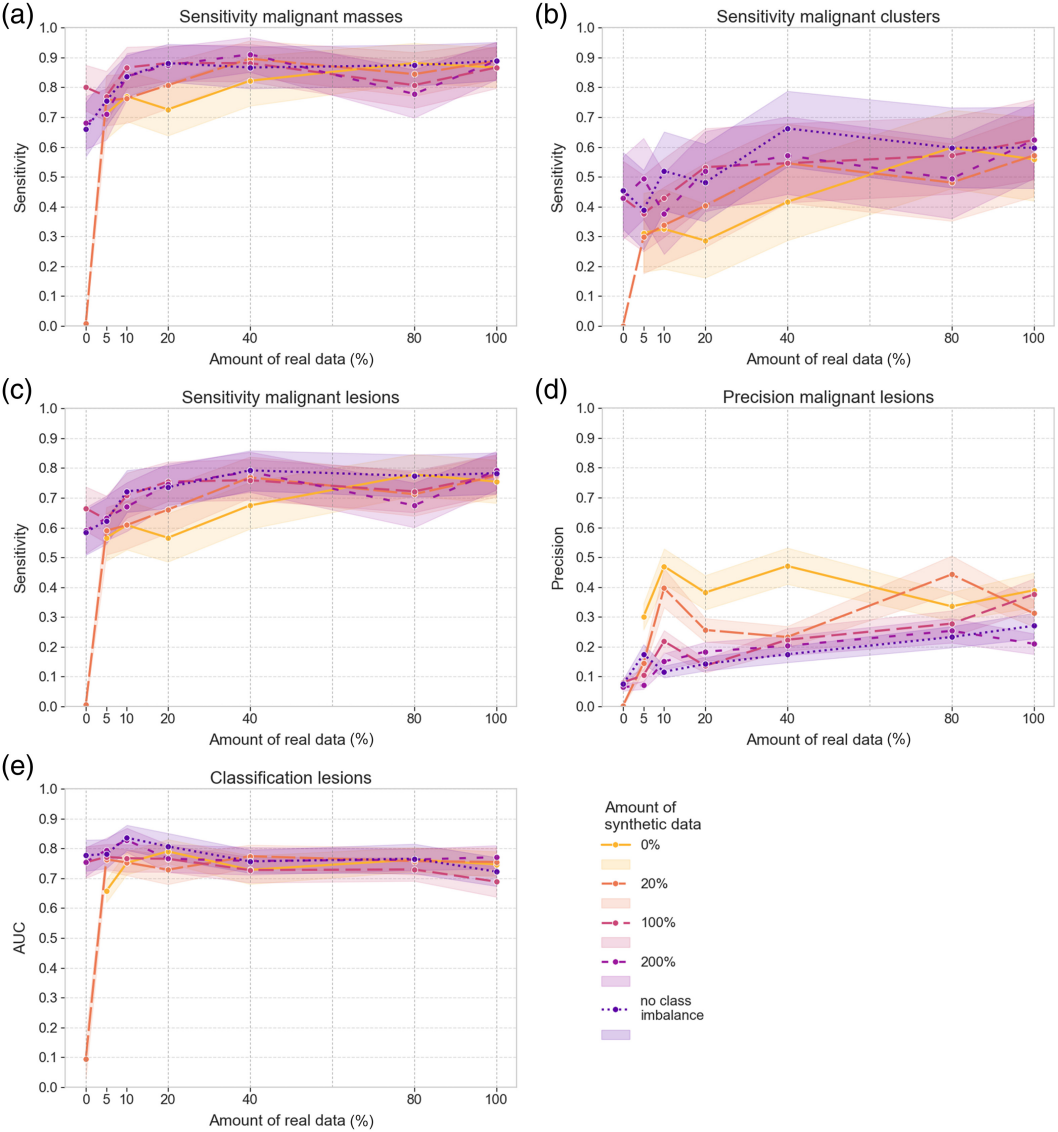
Results on the internal validation set of the DL models trained with different sets of real and/or synthetic data. (a) Detection sensitivity for malignant masses. (b) Detection sensitivity for malignant microcalcification clusters. (c) Detection sensitivity for all malignant lesions. (d) Detection precision for all malignant lesions. (e) AUC for the classification of all lesions.

**Fig. 7:**
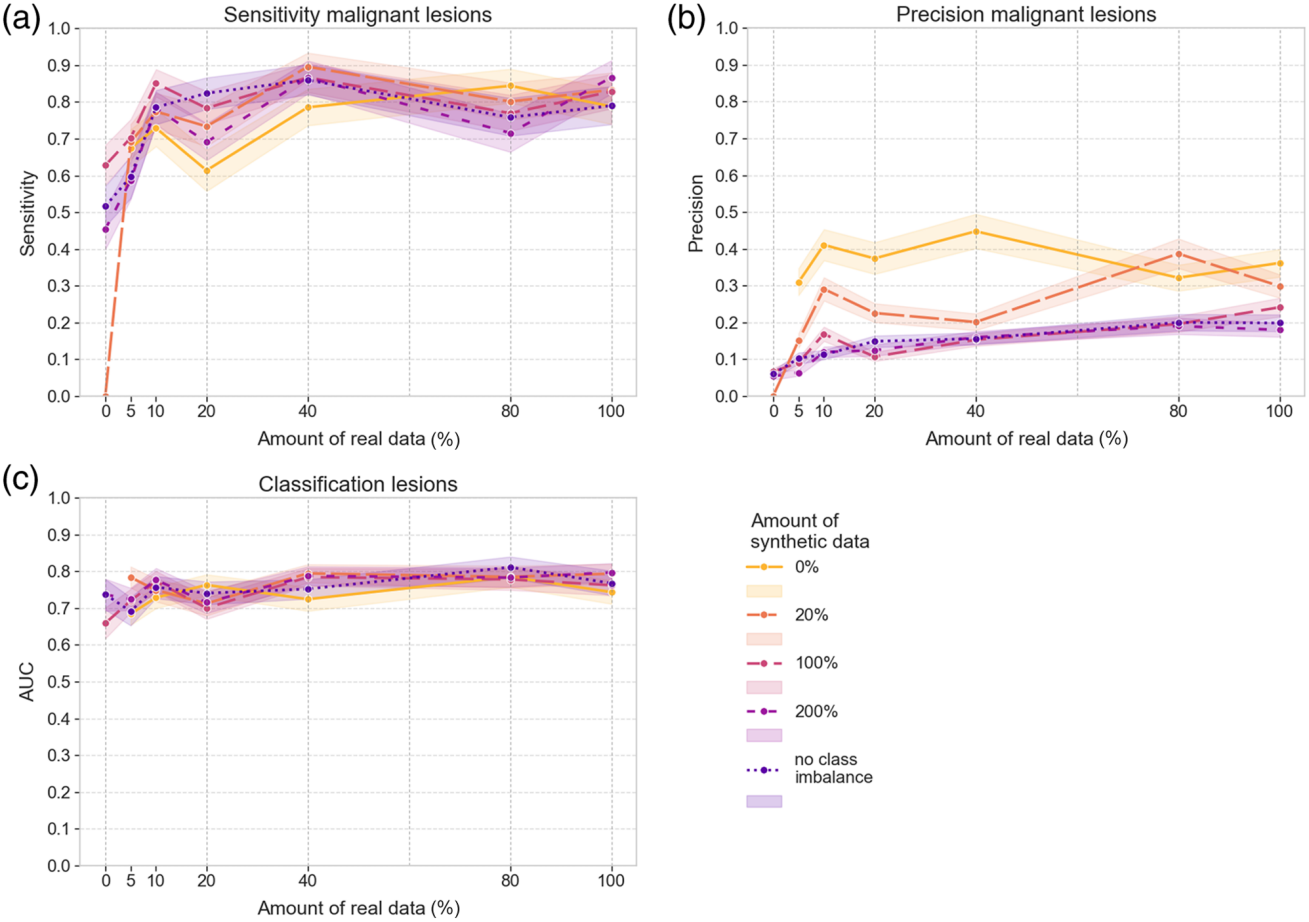
Results on the external validation set of the DL models trained with different sets of real and/or synthetic data. (a) Detection sensitivity for all malignant lesions. (b) Detection precision for all malignant lesions. (c) AUC for the classification of all lesions.

Figures 6 and 7 visualize the results for detecting malignant lesions and classification with the DL model trained in different setups. To study the results for different lesion types, both the combined sensitivity and the sensitivity for either masses [Fig. 6(a)] or clusters [Fig. 6(b)] separately are visualized for the internal validation set. The solid line represents training with real data only. Sensitivity gradually increases as a larger portion of the real training set is included, reaching the highest level of 0.91 for the malignant masses and 0.66 for the malignant clusters. These results indicate lower performance for the detection of small microcalcification clusters, compared with the detection of malignant masses, where a sensitivity of 0.71 is achieved when only 5% of the real training set is used.

The addition of different amounts of synthetic data is indicated by the different dashed lines, and 95% CIs are represented with shaded areas in Figs. 6 and 7. In addition, Supplementals 4 and 5 in the Supplementary Material provide additional values regarding the mean and 95% CIs of the difference between training with solely real data or solely synthetic data and with optimal combinations of real and synthetic data. These results show that synthetic data addition can increase the detection sensitivity of malignant lesions, particularly when less real training data are included. Figures 6(a)–6(c) and 7(a) show that for the 5% to 40% portions of the real training set, adding synthetic data increases the detection sensitivity of malignant lesions to levels similar to those obtained for training with the complete real set. The 95% CIs of the difference of detection sensitivity only contain positive values, showing the addition of synthetic data has a significant positive impact. However, for larger amounts of real training data, this improvement plateaus. In general, there is no strong dependency on the amount of synthetic data used. The highest sensitivity value for malignant lesions is obtained with the training setups incorporating 40% of real training data. For the internal and external validation sets, this value is obtained for the combination with the imbalance-removal and the 20% equivalent synthetic sets, respectively. An additional effect of including synthetic data during training is the drop in precision for malignant lesions, seen for both the internal and external validation set in Figs. 6(d) and 7(b), respectively.

Without real data included, the model trained with the 20% synthetic set failed to detect any malignant lesions. In this case, the difference in detection sensitivity between training with solely synthetic data or the combination of real and synthetic data is high (Tables S7 and S8 and Supplemental 5 in the Supplementary Material). Nevertheless, the other points in Fig. 6(c) show that training with solely synthetic data of simulated clusters can reach a sensitivity of 0.45 for malignant clusters in the internal validation set without any real case present in the training set. In addition, they reach a sensitivity of 0.80 for malignant masses [Fig. 6(a)] even though no mass was yet included during training, as well as an overall sensitivity of 0.63 for any malignant lesion in the external validation set [Fig. 7(a)]. These high levels of sensitivity come again at the cost of a low level of precision, with AUC values for training with synthetic data reaching only 0.78 and 0.74 on the internal and external validation sets, respectively.

The change in AUC with added synthetic data in Figs. 6(e) and 7(c) is less significant compared with the change in detection sensitivity and precision, as can be seen by the overlying curves and 95% CIs. Similarly, the 95% CIs of the AUC difference in Supplemental 4 in the Supplementary Material show both negative and positive values, revealing no consistent increase or decrease. AUC values remain around 0.75 overall, with a division of 0.8 for the classification of masses and around 0.5 for the classification of clusters in the internal validation set. This means that for clusters, the performance of the DL model is at the guessing level, which is related to the large number of falsely detected ROIs of calcifications. As we considered the ground truth class of a falsely detected lesion ROI to be benign, all these ROIs with a predicted malignant label will also be wrongly classified, causing a drop in the classification performance. As the number of clusters in the internal validation set is smaller than the number of masses, the number of falsely detected ROIs will also have a larger impact on the AUC of benign versus malignant cluster classification.

#### 3.3.3 Ensembled model performance

The handcrafted radiomics classifier trained on the complete real training set obtained AUC values of 0.90 (95% CI: 0.86 to 0.94) and 0.84 (95% CI: 0.79 to 0.88) on the real lesions delineated by the radiologist in the internal and external validation set, respectively. The distinction of lesion types in the internal validation set reveals AUC values of 0.93 (95% CI: 0.90 to 0.96) and 0.73 (95% CI: 0.61 to 0.85) for masses and clusters, respectively.

**Fig. 8:**
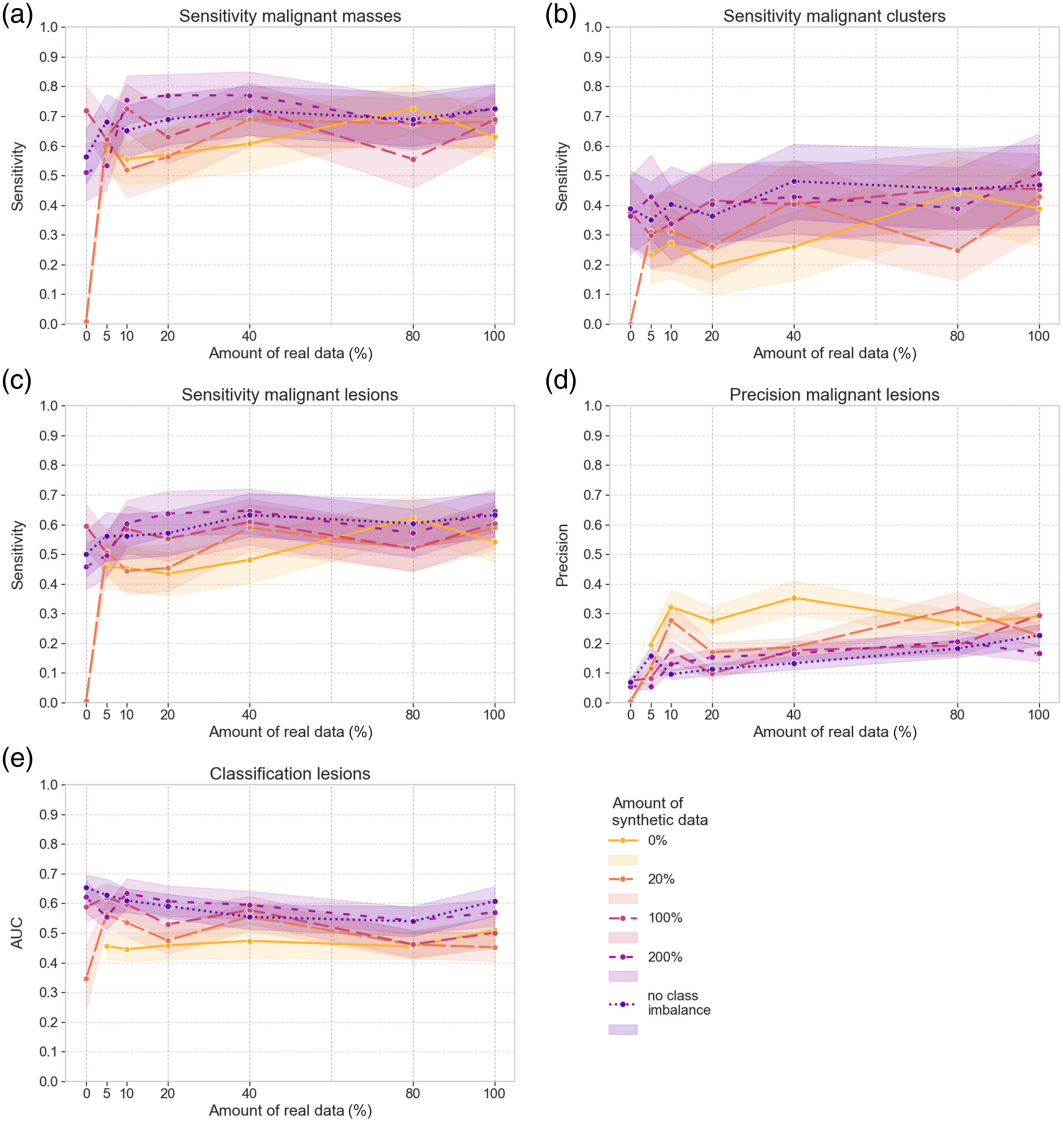
Results on the internal validation set of the ensembled models of the DL models trained with different sets of real and/or synthetic data and the handcrafted radiomics model. (a) Detection sensitivity for malignant masses. (b) Detection sensitivity for malignant microcalcification clusters. (c) Detection sensitivity for all malignant lesions. (d) Detection precision for all malignant lesions. (e) AUC for the classification of all lesions.

**Fig. 9:**
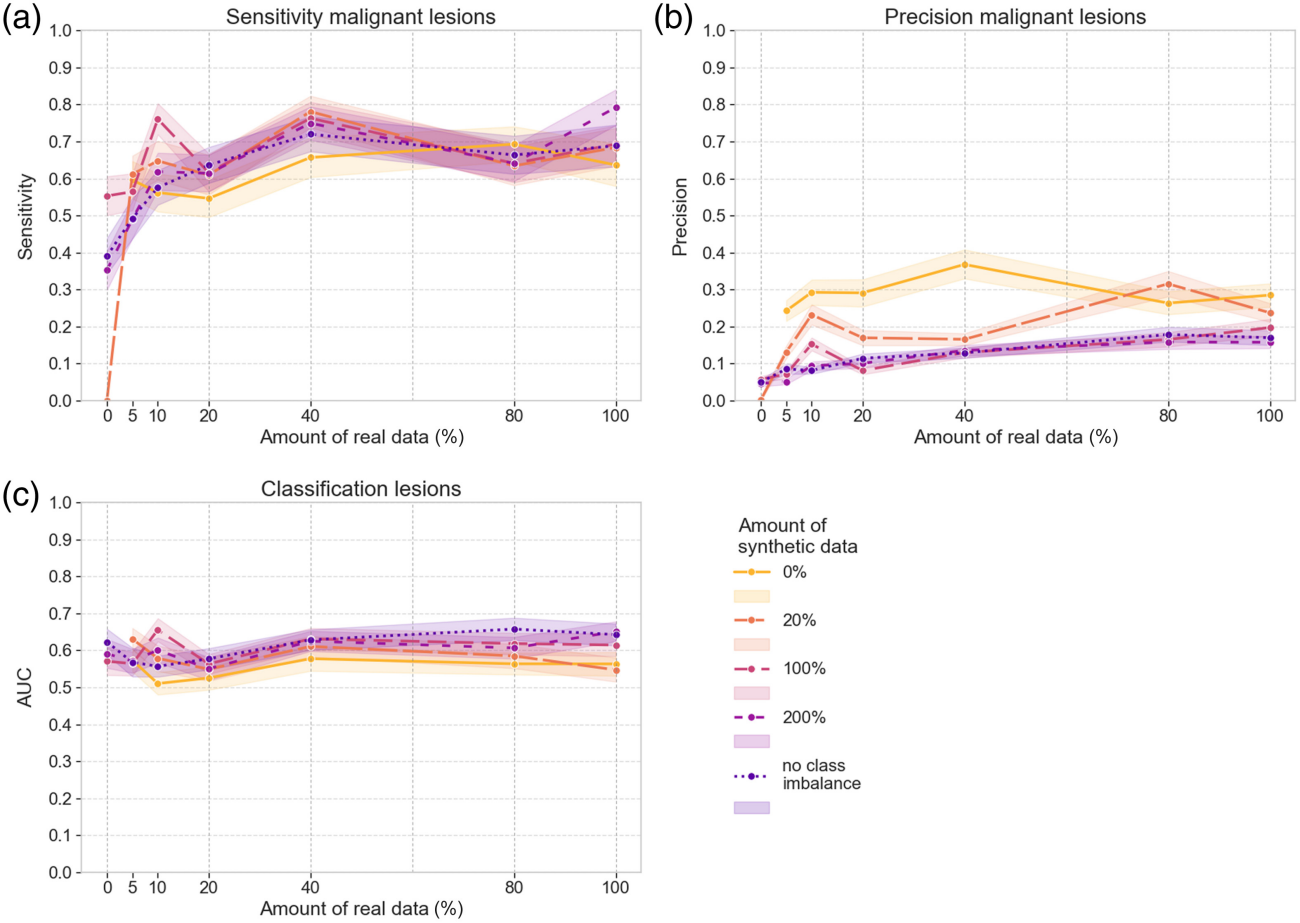
Results on the external validation set of the ensembled models of the DL models trained with different sets of real and/or synthetic data and the handcrafted radiomics model. (a) Detection sensitivity for all malignant lesions. (b) Detection precision for all malignant lesions. (c) AUC for the classification of all lesions.

The curves in Figs. 8 and 9 show the results when the class prediction of the handcrafted radiomics classifier is combined with that of the DL model for all the ROIs returned by the DL model in the detection stage. As the probability values are averaged, this might lead to a different predicted class and can therefore also impact the detection sensitivity and precision. Overall, the graphs show reduced performance for the ensembled model compared with the DL model alone. Although the detection sensitivity of malignant lesions in the external validation set fluctuated around 0.8 for the DL model, the values for the ensembled model lie around 0.7 [Fig. 9(a)]. The highest sensitivity levels for detecting malignant lesions are obtained at the combination of 40% real training data with 200% equivalent synthetic data for the internal validation set and at the combination of 100% real training data with 200% equivalent synthetic data for the external validation set. AUC also drops to ∼0.6. One reason for this might be that the handcrafted radiomics model has only been trained with true lesion ROIs and might therefore have difficulties correctly interpreting the falsely detected ROIs predicted by the DL model. Also, when averaging the probability values of both models, a wrong label with higher probability might overrule the other and thus also potentially affect classification performance as compared with the standalone DL model.

Adding synthetic data again introduced more falsely detected ROIs and thus lowered the precision with a larger reduction in the AUC in Fig. 8(e). For this ensembled model, the 95% CIs of training with real data show less overlap with those obtained when training with a combination of real and synthetic data, as compared with the DL model’s predictions alone. This is best visible for the lowest amounts of real data, 5% to 20%. This is also confirmed by the 95% CIs of the AUC difference in Tables S5 and S6 in the Supplementary Material: most 95% CIs only contain positive values, with mean differences in AUC up to 0.19 and 0.15 for the internal and external validation set, respectively. For the 40% to 100% real datasets, differences are smaller and occasionally negative, which means there is no significant impact.

#### 3.3.4 Case analysis

**Fig. 10:**
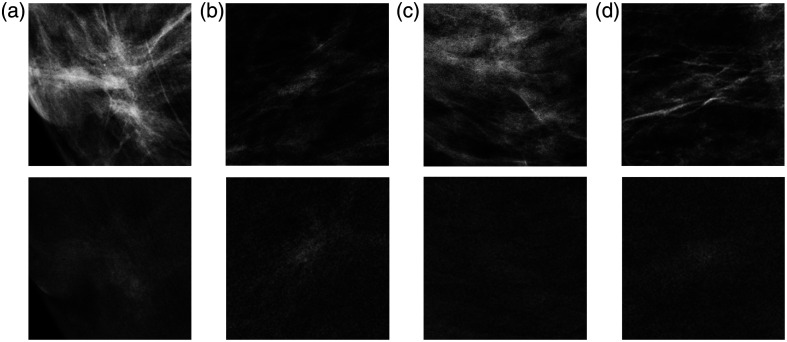
Case examples of the external validation set with the low-energy image in the upper line and the recombined image in the lower line. (a) and (b) True malignant lesions, not detected by the DL models. (c) and (d) No suspicious regions but are falsely detected as a malignant lesion by at least one of the DL models.

**Fig. 11:**
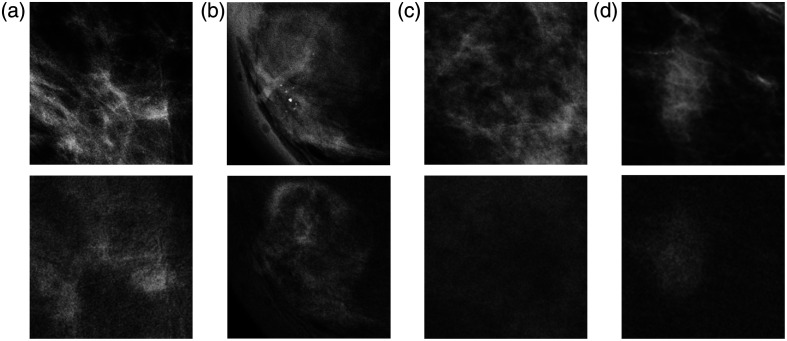
Case examples of the external validation set with the low-energy image in the upper line and recombined image in the lower line. (a) and (b) True malignant lesions, with different classification predictions by either the DL model or the handcrafted radiomics model. (c) and (d) No suspicious regions but falsely detected as a malignant lesion by either the DL or the ensembled model.

To analyze some specific outputs of the predictive models, Figs. 10 and 11 contain example patches from the external validation set. As the highest levels of sensitivity for malignant lesion detection were observed at the 40% real training subset, we consider the predictions of the DL model made with this set. In addition, the predictions of the DL model made with the combination of this set and the imbalance-removal synthetic set are analyzed.

Cases (a) and (b) of Fig. 10 both consist of a true malignant lesion present, not detected by the DL model trained with 40% of real data. The lesion in Fig. 10(b) was correctly characterized as a malignant lesion following the addition of synthetic training data, suggesting a positive impact. In contrast, the case in Fig. 10(a) was still not detected by the DL model when trained with the combination of real and synthetic data.

Although increasing the fraction of malignant lesions detected, adding synthetic data also increased the number of falsely detected ROIs. Cases (c) and (d) of Fig. 10 show regions not annotated by the radiologist as being suspicious. The DL model trained with real data only did not mark case (c) as suspicious, whereas the DL model trained with the real synthetic combination, indicated a malignant cluster. Case (d) shows a falsely detected malignant lesion for both DL models; the real data-trained model assigned a prediction score of 0.23 for a malignant mass, whereas the combination-trained model assigned a prediction score of 0.79 for a malignant cluster thus indicating higher certainty about the presence of a lesion at this location.

Some examples of the predictions of the ensembled model discussed in Sec. 3.2.3 are visualized in Fig. 11. These examples all resulted in a dubious label, denoting that the DL and the handcrafted radiomics model each predicted a different class. Cases (a) and (b) were both annotated as malignant lesions, yet the predictive models disagreed. The DL model predicted Fig. 11(a) to be a benign mass, whereas the handcrafted radiomics classifier predicted a high malignant score, which resulted in a malignant score when averaged in the ensembled model. Figure 11(b) depicts a contrary example, where the DL model correctly predicted a malignant lesion. The handcrafted radiomics model gave a high benign score, and as a result, the ensembled prediction incorrectly characterized this as a benign lesion, resulting in a false negative case.

Figure 11(c) shows a non-suspicious region as annotated by the radiologist but falsely predicted to be a benign lesion by the DL model. The ensembled model changed this classification, giving a false malignant lesion detection. Although no suspicious lesion was annotated for Fig. 11(d), the DL model predicted a malignant lesion, and although the handcrafted radiomics model gave a higher score to a benign lesion, the average score of the ensembled model decision still resulted in a falsely detected malignant lesion.

## 4 Discussion

### 4.1 Synthetic Data Generation

This work presented a method to create synthetic CEM cases with simulated microcalcification clusters and enhancement inserted in real cases. This automated pipeline provided the means to generate a large set of cases applicable for DL model training. Previous work on generating synthetic breast images and breast lesions has predominantly focused on DM imaging. Several methods for breast lesion modeling and insertion with either hybrid or total simulation frameworks exist,^30^ although it is not common to consider the specific image as a starting point when generating a breast lesion model I. For example, Plourde et al.^31^ considered background intensity to build a computational growth model capable of simulating a cluster at a specific location in a DM image. Although Plourde et al. only considered local intensity, our method incorporates a larger set of radiomics features for choice of both location and candidate calcification regions.

Recent approaches increasingly utilize generative adversarial networks (GANs) or diffusion models for synthetic image generation, often aiming to enhance DL models for classification,^32^^,^^33^ segmentation,^34^ or detection^35^^,^^36^ by enlarging the training set. Most of these approaches provide ROIs, whereas the detection task in particular requires the generation of full high-resolution mammograms. Therefore, Shen et al.^36^ implemented and trained an infilling GAN to insert masses into DM images, transforming lesion-free patches or mammograms into images with lesions. This pipeline more closely resembles the approach of this study, which inserts the microcalcification clusters and enhancement in lesion-free images. Both methods benefit from the availability of images with real breast textures eliminating the need to synthesize those, but still facing the problem of smoothly blending the simulated lesion with the surrounding tissue.

The enhancement simulation in the recombined image described in this study is a new technique, which can be attributed to the relatively recent clinical adoption of CEM, and the scarcity of available CEM datasets and quantitative enhancement values. Analysis of the recombined image and enhancement patterns remains difficult due to the proprietary nature of the recombination algorithm employed by the vendors. As a result, some assumptions regarding the enhancement level had to be made. Effects such as artifacts (e.g., breast-in-breast border) and background parenchymal enhancement could be taken into account. The simulation of enhancement and microcalcification clusters were both based on the lesions that were not detected by the DL model trained solely on real data. Both of these simulations followed a distinct pipeline and one aspect did not consider the unique characteristics of the other. One potential means of improving the simulated data in the future might be to correlate the appearance of the same lesion in the low-energy and the recombined images.

### 4.2 Predictive Model Performance

This work investigated a DL model’s performance to detect and classify breast lesions in CEM. The re-implementation of the previously successful mask R-CNN network resulted in a maximum detection sensitivity of 0.90 (95% CI: 0.86, 0.93) for malignant lesions and 0.80 (95% CI: 0.77, 0.827) for all lesions in the external validation set for this study. Beuque et al.^16^ had reported a value of 0.90 for all lesions, however without mentioning precision or false positive rates. The highest AUC found in this study was 0.81 (95% CI: 0.78, 0.84), whereas previous studies have described values up to 0.97.^15^ One reason for the lower AUC might be due to the simultaneous incorporation of detection and classification in the model used in this study, instead of only classifying pre-segmented lesion patches.^15^^,^^37^ The performance of the current model can therefore be degraded by falsely detected ROIs. In addition, this study also considered the classification at the ROI level, rather than at the image or patient level. Although the latter option applies in a screening setting to determine which patients require follow-up, classifying the suspicious finding explicitly is relevant when determining whether a biopsy is required or not. As CEM is primarily applied in the follow-up of patients with suspicious findings, we consider this way of classification is of greater use to the radiologist tasked with deciding which specific lesions are of concern and may require biopsy.

As with many clinical datasets, available CEM cases with corresponding annotations are limited, especially for rare types of breast lesions. This study hypothesized that adding synthetic data to the training set of a DL model would enhance the performance, which was partially validated. An improvement in the detection sensitivity of malignant lesions was observed when smaller real sets were used. Similar results were observed for mass detection on digital mammograms with synthetic data generated with either Monte Carlo–based simulations^38^ or with GANs.^35^^,^^36^ However, above an amount of 40% of the real training set, adding synthetic data had no or limited significant impact on the sensitivity. This plateau is also seen when real cases are used in the training set and may reflect the ability of the model to extract relevant features for the detection and characterization tasks. These results suggest that even though synthetic data can be beneficial, its applicability to improve DL models has its limitations. Although small training sets can benefit from the augmentation with synthetic data, larger sets might already incorporate the distribution required to obtain optimal results.

One interesting aspect is that a DL model trained with solely synthetic data of a specific type (in this case difficult-to-detect microcalcification clusters) can achieve similar performance to a model trained with real data, and can even generalize to detect other lesion types (in this case malignant masses) as well. This raises ideas for future investigations on the requirements of data to train and fine-tune a DL model. Pre-training with existing weights might influence this, pointing toward the potential of foundation models which require only minimal adjustment with a smaller dataset for a specific task.^39^^,^^40^

Beyond the limitations in the data, it is still necessary to address the limitations in the model setup. To ensure a fair comparison and to ensure only the impact of adding synthetic data was assessed, none of the DL model’s parameters or architecture was modified. However, training the model with different datasets may yield another optimal model setting. In addition, the data imbalance should be further addressed. As the validation set was never altered, the microcalcification clusters were in the minority, and optimal model selection might have primarily relied on mass features. Although these training and validation sets were representative of the population and lesion type distribution, future work could explore whether changing the data distribution could improve optimal performance and robustness on external data. Furthermore, it could be investigated whether the handcrafted radiomics classifier could also benefit from the addition of synthetic data in its training data or from incorporating four classes, similar to the DL model. Although this study already included an external validation set to demonstrate the DL model’s performance in a wider setting, evaluation on broader datasets, possibly from multiple centers and multiple vendors, would be of further interest. Validation could also be extended to sets comprising images of breasts without suspicious findings as well, which could possibly change final outcomes regarding the performance of the models.

## 5 Conclusion

This work has introduced a method to create synthetic cases of CEM data. Image-specific microcalcification clusters were modeled considering the textures in the low-energy images of lesion-free breasts. In addition, enhancement was simulated at a corresponding location in the recombined image considering enhancement values typically found for real lesions.

The hypothesis that adding synthetic data during training of a DL model can improve performance was partially confirmed. For smaller sets of real data, detection sensitivity was increased, although at the cost of a drop in precision. Especially noteworthy was the relatively high number of malignant lesions that could be detected without any real case present in the training set, confirming the utility of synthetic data. These findings demonstrate the potential of synthetic data for predictive model training, especially when real data are scarce or imbalanced. In these cases, (tailored) synthetic images can serve as a helpful tool, provided model setup and data distribution are optimized.

## Supplementary Material

10.1117/1.JMI.12.S2. S22006.s01

## Data Availability

The clinical data analyzed during this study are not publicly available due to ethical concerns. The other data that support the findings of this article are available from the corresponding author by request. The code used to generate the results is available in a GitHub repository at https://github.com/precision-medicine-um/real_synthetic_for_CEM.
