# Supplementary material for: Impact of synthetic data on training a deep learning model for lesion detection and classification in contrast-enhanced mammography
Source: J Med Imaging (Bellingham). 2025 Apr 28;12(Suppl 2):S22006. doi: 10.1117/1.JMI.12.S2.S22006 (PMC12036226; doi:10.1117/1.JMI.12.S2.S22006)
Supplement: Supplementary file 1 [file JMI_012_S22006_SD001.docx]

# Supplemental 1 : Enlarging smoothing method

The method developed to smoothen the sharp corners of the convex hull, is based on Chaikin’s corner cutting algorithm^25^. Instead of cutting the corners, which reduces the size of the shape, a similar method enlarges the shape by adding more points and edges on the outside.

Initially, the convex hull is represented as a polygon defined by $n$ vertices $\left\{ P_{i} \right\}_{i=1}^{n}$ and $n$ edges joining the consecutive points (Figure S1). The last edge joins$P_{n}$ and $P_{1}$, thus $i-1$ will be defined as $n$ for $i=1$. With the implemented technique this initial polygon is smoothed by adding more vertices.

First, the technique creates two neighbor points for each vertex on both adjacent edges. These lie at a distance $f*l$ from the respective vertex, defined by a factor $f$ between 0.0 and 1.0, and the length $l$ of the respective edge. For a vertex $P_{i}$ the neighbor points can be defined as $S_{ii-1}$ on the edge connecting $P_{i}$ and $P_{i-1}$, and as $S_{ii+1}$ on the edge connecting $P_{i}$ and $P_{i+1}$. Neighbor points are then connected by drawing the lines through $S_{ii-1}$ and $S_{ii+1}$ for every $i$.

Next, a line is drawn through each $P_{i}$ parallel to the line connecting its neighbor points. Wherever this parallel line through $P_{i}$ intersects with the neighbour point lines of the previous and next vertices, intersection points are defined. $Q_{ii-1}$ is the intersection with the line through $S_{i-1i}$ and $S_{i-1i-2}$, and $Q_{ii+1}$ is the intersection with the line through $S_{i+1i+2}$ and $S_{i+1i}$. This new set of points$\left\{ Q_{ii-1},Q_{ii+1} \right\}_{i=1}^{n}$ define the vertices of a new polygon surrounding the original polygon with more smoother corners.

Figure S1 visualizes the method in which a polygon is smoothly enlarged to a new polygon for various values of factor $f$. This process can be repeated to increase the smoothness of the shape.

Chaikin GM. An algorithm for high-speed curve generation. Comput Graph Image Process. 1974;3(4):346-349. doi:10.1016/0146-664x(74)90028-8
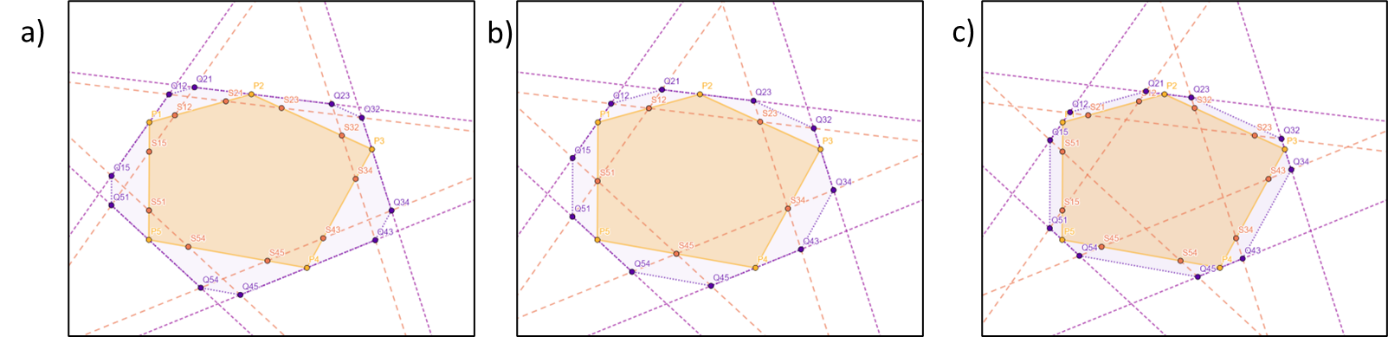


Figure S1: Examples of the enlarging smoothing method for a) d=1/4, b) d=1/2 and c) d=3/4. The original polygon has full edges and the enlarged polygon has dashed edges.

# Supplemental 2: Training data setups

The values in Table S1 indicate the size and proportion of each training data setup. Note that selection and combination was performed on a patient level, but due to some missing images or information, not all image sets are exactly equal to twice the number of patients.

Table S1: Amount of images in each training dataset

| Amount of real data | Amount of synthetic data | Total number of images | Benign without calcifications | Malignant without calcifications | Benign with calcifications | Malignant with calcifications |
| --- | --- | --- | --- | --- | --- | --- |
| 0% | 0% |  |  |  |  |  |
|  | 20% | 63 | 0 | 0 | 18 | 45 |
|  | 100% | 327 | 0 | 0 | 93 | 234 |
|  | 200% | 650 | 0 | 0 | 187 | 463 |
|  | No imbalance | 1034 | 0 | 0 | 296 | 738 |
| 5% | 0% | 80 | 37 | 36 | 2 | 5 |
|  | 20% | 143 | 37 | 36 | 20 | 50 |
|  | 100% | 407 | 37 | 36 | 95 | 239 |
|  | 200% | 730 | 37 | 36 | 189 | 468 |
|  | No imbalance | 1114 | 37 | 36 | 298 | 743 |
| 10% | 0% | 168 | 71 | 71 | 10 | 16 |
|  | 20% | 231 | 71 | 71 | 28 | 61 |
|  | 100% | 495 | 71 | 71 | 103 | 250 |
|  | 200% | 818 | 71 | 71 | 197 | 479 |
|  | No imbalance | 1202 | 71 | 71 | 306 | 754 |
| 20% | 0% | 326 | 139 | 116 | 19 | 52 |
|  | 20% | 389 | 139 | 116 | 37 | 97 |
|  | 100% | 653 | 139 | 116 | 112 | 286 |
|  | 200% | 976 | 139 | 116 | 206 | 515 |
|  | No imbalance | 1360 | 139 | 116 | 315 | 790 |
| 40% | 0% | 658 | 285 | 260 | 34 | 79 |
|  | 20% | 721 | 285 | 260 | 52 | 124 |
|  | 100% | 985 | 285 | 260 | 127 | 313 |
|  | 200% | 1308 | 285 | 260 | 221 | 542 |
|  | No imbalance | 1692 | 285 | 260 | 330 | 817 |
| 80% | 0% | 1319 | 572 | 503 | 65 | 179 |
|  | 20% | 1382 | 572 | 503 | 83 | 224 |
|  | 100% | 1646 | 572 | 503 | 158 | 413 |
|  | 200% | 1969 | 572 | 503 | 252 | 642 |
|  | No imbalance | 2353 | 572 | 503 | 361 | 917 |
| 100% | 0% | 1644 | 702 | 626 | 92 | 224 |
|  | 20% | 1707 | 702 | 626 | 110 | 269 |
|  | 100% | 1971 | 702 | 626 | 185 | 458 |
|  | 200% | 2294 | 702 | 626 | 279 | 687 |
|  | No imbalance | 2678 | 702 | 626 | 388 | 962 |

# Supplemental 3 : Clinical factors of the MUMC and GR datasets

Table S2 includes an overview of all clinical factors included for the CEM datasets. Most factors were formatted as a choice from a pre-defined list, when available. The GR dataset did not include text from the radiology report.

Table S2: Overview of the clinical factors available for the MUMC and GR datasets.

^1^For the GR dataset there was no description available from the radiology report.

^2^For these clinical factors some data might be incomplete, denoted by ‘not reported’.

| Clinical factor | Format |
| --- | --- |
| Breast with suspicious lesion present | - Right - Left - Both |
| BIRADS-score | 1 – 6 |
| Radiologist’s description^1^ | Text |
| Size of suspicious finding in millimetre^2^ | Number |
| Biopsy | - Biopsy performed - No biopsy performed |
| Final diagnosis of suspicious lesion | - Ductal carcinoma in situ (DCIS) - Invasive ductal carcinoma (IDC) - Lobular carcinoma in situ (LCIS) - Invasive lobular carcinoma (ILC) - Other carcinoma - Fibroadenoma - Cyst - Negative |
| Grade^2^ | - 0 when not applicable - 1-3 when applicable |
| Estrogen receptor status^2^ | - Negative - Positive |
| Progesterone receptor status^2^ | - Negative - Positive |
| Human epidermal growth factor receptor 2 status^2^ | - Negative - Positive |
| Age | Number |
| Menopause status^2^ | - Premenopause - Perimenopause - Postmenopause |
| Number of pregnancies^2^ | - Number |
| Number of children^2^ | - Number |
| Medication^2^ | - None - Oral contraceptive pill - Hormone replacement therapy |
| Family history of breast cancer^2^ | - No family history - History in first degree |
| Personal history of breast cancer^2^ | - No personal history - Personal history |
| Cup size^2^ | - A - I |

# Supplemental 4: Statistical analysis of impact of synthetic data

In Tables S3 – S6 the results for detection sensitivity and detection precision of malignant lesions, and for classification AUC are analyzed. For each subset of real data the results are compared between training with solely real data and training with the combination of real and synthetic data that could achieve the highest level of detection sensitivity of malignant lesions. The respective set used in this combination is given in the second column. When no real data was included, the results between training with the amount of synthetic training data leading to the lowest level of detection sensitivity of malignant lesions and with the amount of synthetic data leading to the highest level are compared.

Each cell holds the difference in levels obtained by the combination set and the real set, with the 95% confidence intervals (CI) of bootstrapping given between brackets. A 95% CI comprising the value 0.0 denotes an overlap between both trained models and therefore no significant impact on performance. Values not denoting a significant impact are denoted in *italic*.

Table S3: Impact of synthetic data on the internal validation set of the DL model.

| Amount of real data | Amount of synthetic data | Difference detection sensitivity  malignant lesions | Difference detection precision malignant lesions | Difference AUC classification |
| --- | --- | --- | --- | --- |
| 0% | 20% - 100% | 0.658  [0.580, 0.732] | 0.078  [0.062, 0.094] | 0.658  [0.570, 0.743] |
| 5% | 200% | *0.063 [-0.052,0.177]* | -0.231 [-0.276, -0.183] | 0.138 [0.083, 0.192] |
| 10% | No imbalance | 0.114 [0.004,0.218] | -0.354 [-0.416, -0.294] | 0.085 [0.028, 0.139] |
| 20% | 100% | 0.189 [0.084,0.294] | -0.246 [-0.310, -0.183] | *-0.025 [-0.082, 0.036]* |
| 40% | No imbalance | 0.117 [0.016,0.220] | -0.297 [-0.363, -0.232] | *0.028 [-0.031, 0.091]* |
| 80% | 0% | *0.0 [0.0,0.0]* | *0.0 [0.0,0.0]* | *0.0 [0.0,0.0]* |
| 100% | 200% | *0.037 [-0.064,0.136]* | -0.181 [-0.251, -0.115] | *0.025 [-0.035, 0.082]* |

Table S4: Impact of synthetic data on the external validation set of the DL model.

| Amount of real data | Amount of synthetic data | Difference detection sensitivity malignant lesions | Difference detection precision malignant lesions | Difference AUC classification |
| --- | --- | --- | --- | --- |
| 0% | 20% - 100% | 0.629  [0.575, 0.680] | 0.066  [0.056, 0.076] | 0.659  [0.617,0.702] |
| 5% | 100% | *0.028 [-0.044, 0.097]* | -0.220 [-0.260, -0.183] | *0.036 [-0.009, 0.080]* |
| 10% | 100% | 0.122 [0.060, 0.183] | -0.242 [-0.289, -0.195] | 0.044 [0.003, 0.085] |
| 20% | No imbalance | 0.210 [0.141, 0.280] | -0.226 [-0.275, -0.182] | *-0.022 [-0.065, 0.021]* |
| 40% | 20% | 0.111 [0.049, 0.174] | -0.247 [-0.300, -0.195] | 0.070 [0.031, 0.111] |
| 80% | 0% | *0.0 [0.0,0.0]* | *0.0 [0.0,0.0]* | *0.0 [0.0,0.0]* |
| 100% | 200% | 0.080 [0.017, 0.145] | -0.242 [-0.282, -0.201] | 0.053 [0.014, 0.093] |

Table S5: Impact of synthetic data on the internal validation set of the ensembled model.

| Amount of real data | Amount of synthetic data | Difference detection sensitivity malignant lesions | Difference detection precision malignant lesions | Difference AUC classification |
| --- | --- | --- | --- | --- |
| 0% | 20% -100% | 0.589  [0.511, 0.664] | 0.069  [0.055, 0.083] | 0.239  [0.119, 0.351] |
| 5% | No imbalance | *0.103 [-0.001, 0.217]* | -0.040 [-0.087, 0.008] | 0.170 [0.097, 0.243] |
| 10% | 200% | 0.150 [0.034, 0.260] | -0.194 [-0.256, -0.135] | 0.189 [0.127, 0.250] |
| 20% | 200% | 0.205 [0.099, 0.308] | -0.121 [-0.177, -0.065] | 0.151 [0.080, 0.221] |
| 40% | 200% | 0.164 [0.054, 0.270] | -0.189 [-0.252, -0.127] | 0.121 [0.046, 0.199] |
| 80% | 0% | *0.0 [0.0,0.0]* | *0.0 [0.0,0.0]* | *0.0 [0.0,0.0]* |
| 100% | 200% | *0.104 [-0.002, 0.201]* | -0.124 [-0.181, -0.067] | *0.060 [-0.007, 0.128]* |

Table S6: Impact of synthetic data on the external validation set of the ensembled model.

| Amount of real data | Amount of synthetic data | Difference detection sensitivity malignant lesions | Difference detection precision malignant lesions | Difference AUC classification |
| --- | --- | --- | --- | --- |
| 0% | 20% -100% | 0.553  [0.500, 0.605] | 0.056  [0.047, 0.065] | 0,563  [0.531,0.560] |
| 5% | 20% | *0.015 [-0.055, 0.080]* | -0.112 [-0.144, -0.078] | 0.058 [0.016, 0.100] |
| 10% | 100% | 0.200 [0.136, 0.265] | -0.140 [-0.179, -0.100] | 0.145 [0.103, 0.190] |
| 20% | No imbalance | 0.090 [0.019, 0.162] | -0.177 [-0.215, -0.137] | 0.052 [0.008, 0.096] |
| 40% | 20% | 0.124 [0.053, 0.193] | -0.204 [-0.247, -0.161] | *0.032 [-0.013, 0.075]* |
| 80% | 0% | *0.0 [0.0,0.0]* | *0.0 [0.0,0.0]* | *0.0 [0.0,0.0]* |
| 100% | 200% | 0.156 [0.085, 0.227] | -0.127 [-0.163, -0.092] | 0.088 [0.046, 0.129] |

# Supplemental 5: Statistical analysis of impact of real data

In Tables S7 – S10 the results for detection sensitivity and detection precision of malignant lesions, and for classification AUC are analyzed. For each subset of synthetic data the results are compared between training with solely synthetic data and training with the combination of real and synthetic data that could achieve the highest level of detection sensitivity of malignant lesions. The respective real subset used in this combination is given in the second column. When no synthetic data was included (0% set), the results between training with the amount of real training data leading to the lowest level of detection sensitivity of malignant lesions and with the amount of real data leading to the highest level are compared.

Each cell holds the difference in levels obtained by the combination set and the synthetic set, with the 95% confidence intervals (CI) of bootstrapping given between brackets. A 95% CI comprising the value 0.0 denotes an overlap between both trained models and therefore no significant impact on performance. Values not denoting a significant impact are denoted in *italic*.

Table S7: Impact of real data on the internal validation set of the DL model.

| Amount of synthetic data | Amount of real data | Difference detection sensitivity malignant lesions | Difference detection precision malignant lesions | Difference AUC classification |
| --- | --- | --- | --- | --- |
| 0% | 5%-80% | 0.772  [0.698, 0.840] | *0.034*  *[-0.030, 0.101]* | 0.106  [0.050, 0.162] |
| 20% | 100% | 0.767  [0.694, 0.837] | 0.310  [0.267, 0.355] | 0.661  [0.570, 0.740] |
| 100% | 100% | 0.116  [0.010, 0.217] | 0.296  [0.242, 0.350] | *-0.065*  *[-0.141, 0.011]* |
| 200% | 100% | 0.202  [0.102, 0.300] | 0.145  [0.109, 0.183] | *0.017*  *[-0.044, 0.078]* |
| No imbalance | 40% | 0.208  [0.107, 0.307] | 0.100  [0.070, 0.131] | *-0.020*  *[-0.084, 0.045]* |

Table S8: Impact of real data on the external validation set of the DL model.

| Amount of synthetic data | Amount of real data | Difference detection sensitivity malignant lesions | Difference detection precision malignant lesions | Difference AUC classification |
| --- | --- | --- | --- | --- |
| 0% | 20%-80% | 0.230  [0.154, 0.301] | *-0.054*  *[-0.110, 0.001]* | *0.023*  *[-0.017, 0.065]* |
| 20% | 40% | 0.897  [0.858, 0.932] | 0.201  [0.178, 0.223] | 0.795  [0.770,0.819] |
| 100% | 40% | 0.238  [0.169, 0.304] | 0.087  [0.068, 0.107] | 0.125  [0.078, 0.176] |
| 200% | 40% | 0.413  [0.339, 0.483] | 0.106  [0.086, 0.126] | 0.052  [0.003, 0.104] |
| No imbalance | 20% | 0.307  [0.237, 0.376] | 0.089  [0.071, 0.106] | *0.004*  *[-0.046, 0.054]* |

Table S9: Impact of real data on the internal validation set of the ensembled model.

| Amount of real data | Amount of synthetic data | Difference detection sensitivity malignant lesions | Difference detection precision malignant lesions | Difference AUC classification |
| --- | --- | --- | --- | --- |
| 20%-80% | 0% | 0.191  [0.090, 0.292] | *-0.006*  *[-0.067, 0.058]* | *-0.003*  *[-0.066, 0.062]* |
| 40% | 20% | 0.584  [0.507, 0.660] | 0.183  [0.153, 0.215] | 0.207  [0.087, 0.326] |
| 40% | 100% | *0.015*  *[-0.090, 0.126]* | 0.103  [0.068, 0.138] | *-0.010*  *[-0.078, 0.055]* |
| 40% | 200% | 0.186  [0.072, 0.294] | 0.109  [0.081, 0.137] | *-0.028*  *[-0.102, 0.044]* |
| 40% | No imbalance | 0.132  [0.025, 0.235] | 0.064  [0.037, 0.089] | -0.090  [-0.161, -0.037] |

Table S10: Impact of real data on the external validation set of the ensembled model.

| Amount of real data | Amount of synthetic data | Difference detection sensitivity malignant lesions | Difference detection precision malignant lesions | Difference AUC classification |
| --- | --- | --- | --- | --- |
| 20%-80% | 0% | 0.148  [0.083, 0.216] | *-0.027*  *[-0.074, 0.022]* | *0.039*  *[-0.005, 0.084]* |
| 40% | 20% | 0.781  [0.736, 0.822] | 0.164  [0.148, 0.181] | 0.611  [0.583,0.638] |
| 40% | 100% | 0.210  [0.140, 0.280] | 0.073  [0.056, 0.089] | 0.061  [0.013, 0.110] |
| 100% | 200% | 0.441  [0.369, 0.509] | 0.112  [0.094, 0.131] | 0.056  [0.013, 0.106] |
| 40% | No imbalance | 0.323  [0.265, 0.399] | 0.079  [0.063, 0.096] | *0.005*  *[-0.038, 0.049]* |
